# Investigating the impact of mental rehearsal on prefrontal and motor cortical haemodynamic responses in surgeons using optical neuroimaging

**DOI:** 10.3389/fnhum.2024.1386005

**Published:** 2024-10-21

**Authors:** Hemel N. Modi, Maia Osborne-Grinter, Ronak Patel, Ara Darzi, Daniel R. Leff, Harsimrat Singh

**Affiliations:** ^1^Neuroergonomics and Perception Laboratory, Department of Surgery and Cancer, Imperial College London, London, United Kingdom; ^2^The Hamlyn Centre, Imperial College London, London, United Kingdom

**Keywords:** fNIRS, functional neuroimaging, prefrontal cortex, motor cortex, neuroergonomics, mental rehearsal, surgery, surgical performance

## Abstract

**Introduction:**

Inadequate exposure to real-life operating can impede timely acquisition of technical competence among surgical residents, and is a major challenge faced in the current training climate. Mental rehearsal (MR)—the cognitive rehearsal of a motor task without overt physical movement—has been shown to accelerate surgical skills learning. However, the neuroplastic effect of MR of a complex bimanual surgical task is unknown. The aim of this study is to use functional near-infrared spectroscopy (fNIRS) to assess the impact of MR on prefrontal and motor cortical activation during a laparoscopic knot tying task.

**Methods:**

Twelve surgical residents performed a laparoscopic knot tying task before and after either mental rehearsal (MR, intervention group) or textbook reading (TR, control group). In both groups, fNIRS was used to measure changes in oxygenated hemoglobin concentration (HbO2) in the prefrontal (24 channels) and motor cortices (22 channels). Technical performance was measured using leak volume, objective performance score and task progression score.

**Results:**

MR led to a decrease in HbO_2_ (reduced activation) in the bilateral prefrontal cortex (PFC), and an increase in HbO_2_ (increased activation) in the left middle frontal gyrus, left precentral gyrus, and left postcentral gyrus. No discernible changes in activation were observed after TR in either the PFC or motor cortex. Moreover, smaller ΔHbO2 responses in the right PFC and greater ΔHbO_2_ responses in the left motor cortex were observed in the MR group compared with the TR group. Leak volume was significantly less following MR (*p* = 0.019), but not after TR (*p* = 0.347). Mean objective performance score was significantly higher following MR compared with TR (*p* = 0.043).

**Conclusion:**

Mental rehearsal may enhance surgical skill acquisition and technical proficiency by reducing utilization of attentional resources in the prefrontal cortex and improving neural efficiency in motor areas during a laparoscopic surgical task.

## Introduction

In a craft-based profession like surgery, technical skills have traditionally been acquired through an apprenticeship model of training. Trainees spend many hours in the operating room (OR) initially observing an experienced surgeon, then performing operations under direct supervision, before eventually developing operative independence. However, surgical training faces several challenges which impede trainees from gaining ‘hands on’ experience such as working time restrictions, performance targets imposed on healthcare organizations, and the ongoing conflict between service provision and training (Galasko, 2005; Bates et al., 2007; Ashmore, 2019). More recently, the COVID pandemic during which non-urgent surgical cases were canceled and surgical trainees were re-deployed to work in other departments, has resulted in a significant loss of training in emergency and elective operating (Clements et al., 2021).

Hence, there is a need to develop interventions that can shorten learning curves and expedite surgical skill acquisition. Mental rehearsal (MR) is the cognitive rehearsal of a motor task without overt physical movements (Arora et al., 2011), and may enhance surgeons’ performance by helping them mentally prepare for a procedure, focus their attention on critical steps of an operation, and anticipate potential complications (Anton et al., 2017). Indeed, emerging literature has demonstrated that mental rehearsal can improve surgical performance (Arora et al., 2011; Rao et al., 2015; Cocks et al., 2014; Komesu et al., 2009; Bathalon et al., 2005; Louridas et al., 2015; Anton et al., 2018; Anton et al., 2019), and arguments have been made for its formal integration into the surgical curriculum (Evgeniou and Loizou, 2013).

It is now established that skill acquisition and motor learning are associated with dynamic changes in brain function. The prefrontal cortex (PFC) plays a vital role in executive functions such as working memory, decision-making, and attentional control (Diamond, 2013), and changes in PFC activation have been shown to accompany expertise development and motor learning in surgery (Leff et al., 2007; Leff et al., 2008; Leff et al., 2008). Specifically, the PFC is recruited to a greater extent in novice compared to expert surgeons in whom such tasks have become automated and ingrained (Leff et al., 2007). However, following a period of training and practice, the prefrontal response of the ‘trained’ novices attenuates as performance improves (Leff et al., 2008; Leff et al., 2008).

The primary motor cortex (M1), the supplementary motor area (SMA), and the premotor area (PMA) are responsible for planning and execution of voluntary movements (Halsband and Lange, 2006). Data from functional magnetic resonance imaging (fMRI) (Morris et al., 2015), positron emission tomography (PET) (Duty et al., 2012) and functional near-infrared spectroscopy (fNIRS) (Nemani et al., 2015) experiments during open (Morris et al., 2015) and laparoscopic (Duty et al., 2012; Nemani et al., 2015) tasks depict comparative attenuation of M1 activations amongst expert surgeons compared to novices, implying learning-related movement efficiency is mirrored by efficiencies in motor regions in the brain (Grantcharov et al., 2003; Datta et al., 2002). This suggests that consolidation of skills is associated with greater neural efficiency in motor regions, allowing experts to focus on the finer aspects of motor control as the primary task is more ingrained.

Much of the literature describing the neural processes underpinning mental rehearsal supports the “functional equivalence model” which posits overlapping neural representations in mental rehearsal and physical execution (Moran, 2009; Jeannerod, 2001; Jeannerod, 1995; Kuhtz-Buschbeck et al., 2003; Gerardin et al., 2000; Saiote et al., 2016; Hardwick et al., 2018). An extrapolation of this model would hypothesize that the underlying neurophysiological mechanisms that underpin practice effects would also be comparable (Di Rienzo et al., 2016). To this end, some studies have investigated how mental rehearsal facilitates motor learning and skill acquisition in terms of its effect on learning-dependant brain changes (Di Rienzo et al., 2016; Ladda et al., 2021; Ruffino et al., 2017).

In their early work Pascual-Leone et al. (1995) provided evidence that mental rehearsal induces similar learning-dependant neuroplasticity as physical practice (Pascual-Leone et al., 1995). Using transcranial magnetic stimulation, the authors found that mental rehearsal led to a similar enlargement of cortical representations of hand muscles required for performance of a piano sequence task as was observed with physical practice (Pascual-Leone et al., 1995). Support for cortical reorganization can also be found in studies which used indirect neuroimaging modalities to measure haemodynamic changes in the brain (Lacourse et al., 2004; Nyberg et al., 2006; Jackson et al., 2003; Zhang et al., 2011). Specifically, fMRI studies have shown that mental rehearsal training improves neural efficiency in motor regions by strengthening the cortical representation of the task in primary motor areas, while reducing recruitment of secondary regions (Lacourse et al., 2004; Nyberg et al., 2006). For example, Lacourse et al. (2004) observed increased but more focused activation in the contralateral primary motor cortex and decreased activation in the supplementary and premotor areas with both mental rehearsal and physical training on a button-pressing task (Lacourse et al., 2004). Similarly, Nyberg et al. (2006) demonstrated a contraction in the extent of motor activation following both mental rehearsal and physical practice of a left-handed finger tapping task (Nyberg et al., 2006).

These studies suggest that mental rehearsal training leads to cortical reorganization and improved neural efficiency in motor regions, comparable to the functional changes elicited through physical practice of the same task. However, these studies used simple motor task paradigms [e.g., piano sequence (Pascual-Leone et al., 1995), button pressing (Lacourse et al., 2004), finger movement (Nyberg et al., 2006; Zhang et al., 2011), foot movement (Jackson et al., 2003; Lafleur et al., 2002)] rather than complex bimanual skills required in surgery. In addition, the neuroimaging modalities utilized (e.g., fMRI and PET), would have required subjects to be constrained in the complexity of tasks under study. Utilizing an imaging modality which allows subjects to be freely mobile (e.g., fNIRS) would allow subjects to perform more complex motor tasks. Moreover, the duration of mental rehearsal training in most of the studies ranged from 5 days to 2 weeks (Pascual-Leone et al., 1995; Lacourse et al., 2004; Nyberg et al., 2006; Jackson et al., 2003; Zhang et al., 2011; Lafleur et al., 2002). Such a prolonged period of training would not be feasible in a busy surgical setting. Finally, most of the literature focuses on activation in motor regions. Given the importance of executive function on skills learning, understanding how mental rehearsal impacts PFC activity in addition to that of motor regions would further our understanding of the neural mechanisms of performance improvement with mental rehearsal.

The aim of the current study is to use fNIRS to investigate the impact of mental rehearsal training on prefrontal and motor cortical activation during execution of a complex surgical task (laparoscopic knot-tying). In addition to contributing to the scientific understanding of cognitive rehearsal and its effect on brain behavior, decerning the neural mechanisms by which mental rehearsal improves surgical skill acquisition and retention will provide objective evidence upon which decisions to formally incorporate it into surgical training curricula can be based. The hypothesis is that performance gains arising from mental rehearsal will be underpinned by more focused activation of motor regions indicative of greater neural efficiency, along with attenuated responses in the prefrontal cortex reflecting skill internalization and automaticity.

## Materials and methods

### Subjects

Following ethical approval and after having obtained informed written consent, 12 surgical residents agreed to participate (median age = 31.5 years, 4 females) (Table 1). All subjects were screened for handedness and neuropsychiatric illness (*n* = 0) and were asked to refrain from alcohol and caffeine intake for 24 h prior to participation.

**Table 1:** Subject demographics.

	Mental rehearsal group	Textbook reading group	*p*-value
Number of subjects	6	6	
Mean age (SD)	35.8 (6.7)	31.3 (1.5)	0.163^*^
Male:Female	5:1	3:3	0.545^†^
Mean previous LS experience (SD)^‡^	15.5 (17.4)	8.2 (8.1)	0.372^*^
Median handedness score (range)^§^	66.7 (−25.0–100.0)	100.0 (100.0–100.0)	0.312^‖^

* Independent samples t-test.

† Fisher’s exact test.

‡ Number of times subject has performed a laparoscopic suturing (LS) task.

§ Calculated using the Edinburgh Handedness Inventory.

‖ Mann–Whitney U-test.

### Task paradigm and experimental design

**Figure 1:**
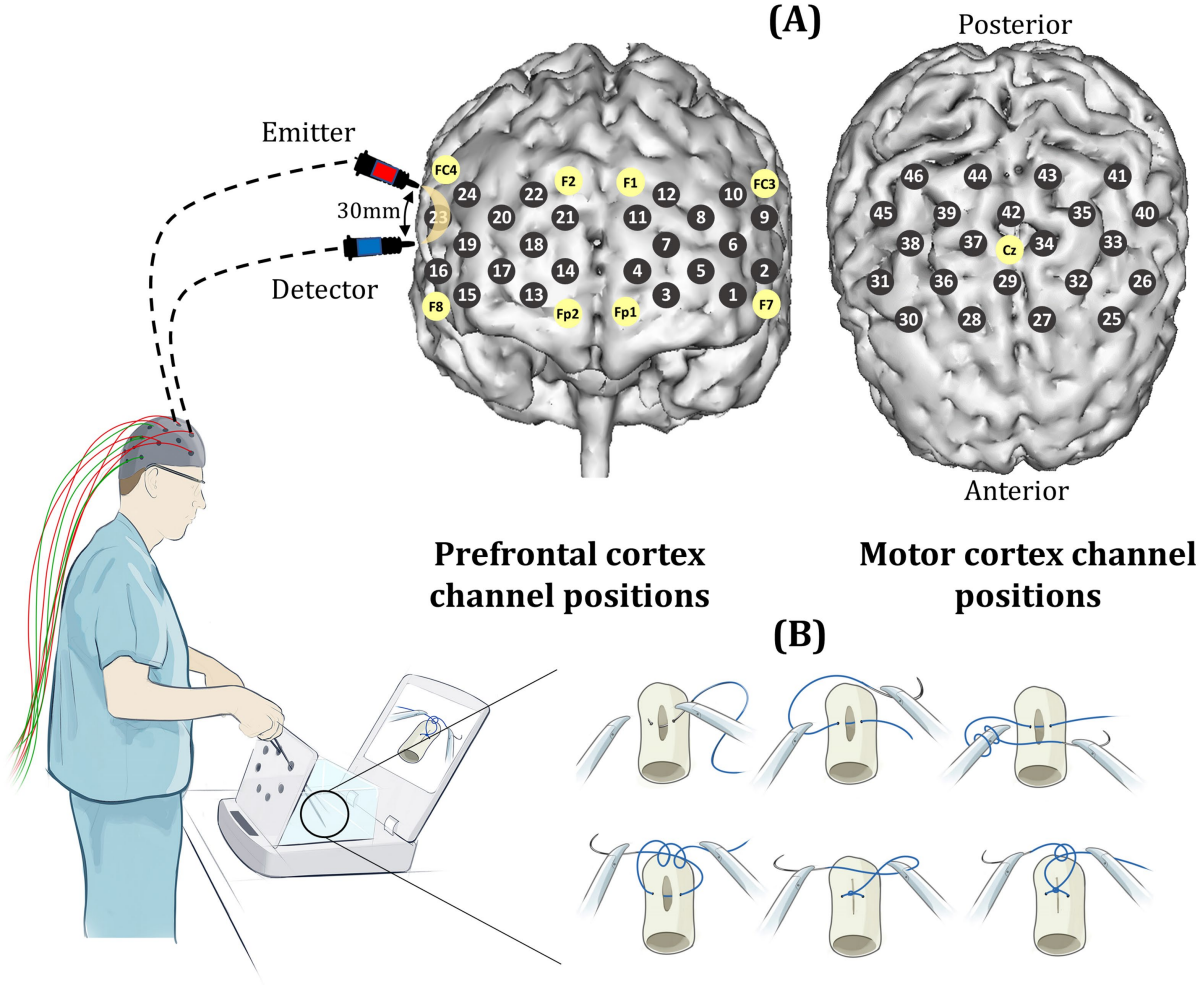
**(A)** Prefrontal and motor channel locations (gray circles) which are positioned according to the international 10–20 system of probe placement (yellow circles). Sources and detectors are separated by an inter-optode distance of 30 mm. **(B)** Laparoscopic knot-tying task performed on a box trainer (iSim2, iSurgicals, UK).

Participants were asked to perform a laparoscopic suturing (LS) task using an intracorporeal technique on a laparoscopic box trainer (iSim2, iSurgicals, UK). The task involved inserting a 2–0 Vicryl^®^ suture (Ethicon, Somerville, NJ, USA) as close to pre-marked entry and exit points on either side of a defect in a Penrose drain. To tie a knot laparoscopically, participants were instructed to formulate one double throw followed by two single throws of the suture (Figure 1).

**Figure 2:**
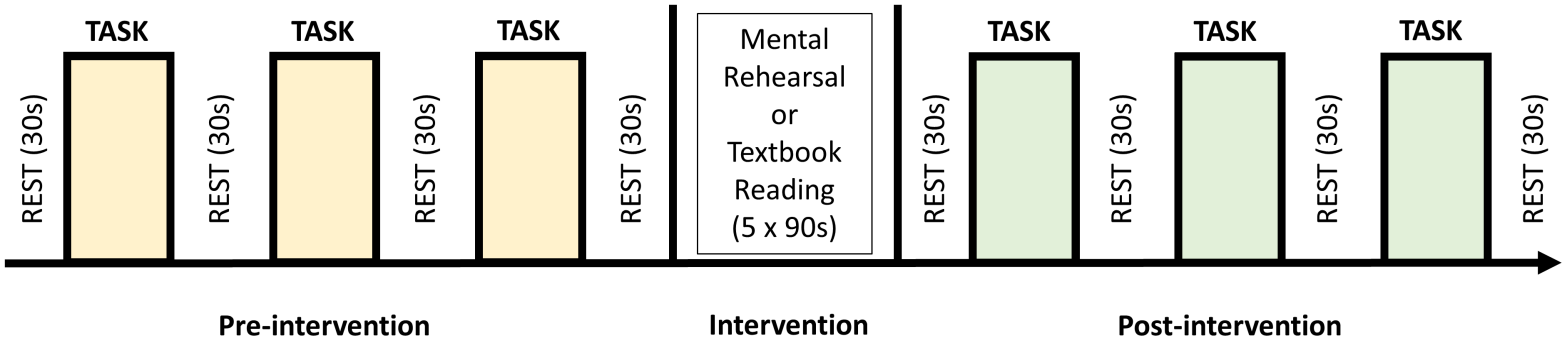
Block study design in which participants performed 3 self-paced trials of the laparoscopic knot-tying task (intertrial rest period of 30 s) before being randomized to undergo five 90-s sessions of either mental rehearsal or textbook reading. This was followed by a further 3 trials of the task.

A block design experiment was conducted in which all participants initially performed the knot-tying task three times (baseline), with an inter-trial rest period of 30-s. Subjects were then randomized into intervention (mental rehearsal [MR]) or control (textbook reading [TR]) groups using a random number generator. Following either MR or TR, participants tied another three knots as described above (Figure 2).

### Control group

Upon completion of the baseline task, participants in the control group read extracts from a paper which outlined the technical steps of the laparoscopic knot-tying task (Croce and Olmi, 2000). The subjects were asked to read the extracts for 90 s and repeated this exercise 5 times.

### Mental rehearsal group

Each participant in the mental rehearsal group listened to a 90-s pre-recorded MR script (Supplementary Text S1). In order to create the script, instructional videos identified the key steps in performing LS as performed by consultant surgeons. Sensory and kinaesthetic sensory cues were identified and included in the script. The script contained not only a list of procedural steps for laparoscopic suturing, but also a vivid description of associated imagery cues to enhance the representation of the task in the subject’s mind and enable them to mentally experience the procedure. Having been reviewed by a consultant surgeon and three trainees, the participants listened to the script five times and were instructed to imagine the steps of the procedure and the associated feelings and sensations, whilst refraining from any physical movement.

### Blinding

One experimenter (RP) was responsible for allocating participants to the MR or TR groups and was therefore not blinded to group allocation. Those involved in data collection and analysis (HNM, MO, and HS) were blinded to group allocation. Furthermore, as participants in both groups were required to perform an activity during the training period (i.e., mentally rehearse a task or read a passage of text), they were unaware as to whether they were allocated to the experimental or control group.

### Outcome measures

#### Brain activation

The ETG-4000 Optical Topography System (Hitachi Medical Co, Japan) was used to measure changes in cortical oxygenated hemoglobin concentrations (HbO_2_) as a marker of functional brain activation across 24 prefrontal and 22 motor cortical channels. Sources and detectors were guided into position based on the international 10–20 system of probe placement (Jurcak et al., 2007), with a source-detector distance of 30 mm (Figure 1). Near-infrared light was delivered at 695 and 830 nm wavelengths.

#### Subjective workload and heart rate

Subjective workload was quantified using the Surgical Task Load Index (SURG-TLX) and STAI-6 questionnaires (Wilson et al., 2011; Marteau and Bekker, 1992). A wireless monitor (Bioharness, Zephyr Technology, USA) continually recorded heart rate (HR). Change in HR from rest to task (ΔHR) was calculated as follows:


$$\Delta HR=\mathrm{Median}\;HR\;\left(\mathrm{Task}\right)-\mathrm{Median}\;HR\;\left(\mathrm{Rest}\right)$$

#### Technical performance

Technical skill was objectively assessed using three parameters, summarized as follows:

*Task Progression Score (TPS; arbitrary units, au)*: Each task episode was assigned a score based on task progression, with 1 point awarded for each of the following steps: (Galasko, 2005) mounting the needle onto the needle holder, (Bates et al., 2007) needle insertion into the drain, (Ashmore, 2019) exiting the needle from the drain, (Clements et al., 2021) double throw, (Arora et al., 2011) 1st single throw, and (Anton et al., 2017) 2nd single throw of a laparoscopic reef knot. The TPS comprised the total number of points obtained during the task (maximum score = 6).

*Objective Performance Score (OPS; arbitrary units, au)*: Adapted from the FLS scoring system for LS (Ritter and Scott, 2007), and utilized by several previous authors as a valid method of combining raw FLS performance measures into a single composite score (Anton et al., 2019; Stefanidis et al., 2017; Korndorffer et al., 2005; Stefanidis et al., 2007; Prabhu et al., 2010), the OPS was calculated as follows for each knot tied:


$$\begin{array}{l} OPS=\\ \left[\begin{array}{l} \mathrm{Maximum}\;\mathrm{permissible}\;\mathrm{time}\;for\;\mathrm{task}\;\mathrm{completion}\;\left(300s\right)-\\ \mathrm{Time}\;\mathrm{taken}\;for\;\mathrm{knot}\;\mathrm{completion}\;\left(s\right)-\left(10\;x\;\mathrm{error}\;\mathrm{score}\;\left(\mathrm{mm}\right)\right)\\ -\left(100\;x\;\mathrm{knot}\;\mathrm{failure}\;\mathrm{score}\;\left(au\right)\right) \end{array}\right] \end{array}$$

The error score indicates the accuracy of needle placement and is calculated as follows:


$$\begin{array}{l} \mathrm{Error}\;\mathrm{Score}=\\ \left[\begin{array}{l} \mathrm{Distance}\;\left(\mathrm{mm}\right)\;\mathrm{between}\;\mathrm{needle}\;\mathrm{insertion}\;\mathrm{point}\;\mathrm{and}\;\\ \mathrm{premarked}\;\mathrm{target}\;\mathrm{position}+\mathrm{Distance}\;\left(\mathrm{mm}\right)\;\mathrm{between}\;\\ \mathrm{needle}\;\mathrm{exit}\;\mathrm{point}\;\mathrm{and}\;\mathrm{premarked}\;\mathrm{target}\;\mathrm{position} \end{array}\right] \end{array}$$

The knot failure score represents the strength of the tied knot. Knot slippage is allocated a score of 1, and knot breakage is given a score of 2.

*Leak Volume (LV; ml)*: Saline was infused through each drain at a rate of 150 drops/min controlled via a digital pump. The volume of saline leaking from the closed defect over a 1-min period was recorded to assess the quality of defect closure.

#### Mental imagery ability

Mental imagery was assessed using a validated Mental Imagery Questionnaire (MIQ) adapted from Cumming et al. (2007) and which has been validated for use in surgery (Arora et al., 2010). Subjects completed the MIQ before and after receiving the MR or control intervention. The MIQ enabled the quality of mental imagery experiences to be quantified. The MIQ is an 8-item questionnaire, on which each item is scored on a 1–7 Likert scale. The items in the questionnaire assessed mental readiness (q1), confidence in performing the task (q2 and q3), usefulness of MR in task preparation (q4), quality of visual imagery (q5 and q6), kinesthetic imagery (the cognitive re-creation of the feeling of movement) (q7), and knowledge of the technical aspects of the task (q8) (Supplementary Figure S1).

### Data processing and statistical analysis

Statistical analysis was performed using SPSS version 23.0 (IBM Corp., Armonk, NY, USA). A threshold *p* < 0.05 was set as the threshold for statistical significance.

#### Stress, technical skills data and mental imagery ability

Within-group (i.e., pre-*vs* post-intervention) comparisons were analyzed using the paired samples t-test for parametric data (i.e., OPS, leak volume, SURG-TLX and MIQ) and the Wilcoxon Signed Ranks test for non-parametric data (i.e., heart rate, progression score and STAI-6). Between-group (i.e., MR vs. TR) comparisons were analyzed using the independent samples t-test (parametric data) or the Mann–Whitney U test (non-parametric data) to determine significant between-group differences in stress, performance and mental imagery ability.

#### Functional neuroimaging data

Functional neuroimaging data was pre-processed using a bespoke MATLAB-based toolbox (HOMER2) (Huppert et al., 2009). Data quality checks were performed with the standard functionality using the function ‘hmrenPruneChannels,’ with a standard deviation of 0–45 dB and a signal to noise ratio threshold of 2 a.u. Channels which exhibited very low optical intensities (<1) were excluded. High frequency noise and electrocardiographic effects were minimized using a low-pass filter (0.5 Hz). Across the population group (552 channels), 16 channels were excluded due to poor optical signals (data rejection rate of 2.9%). Raw mean intensity values were converted to changes in optical density relative to the mean of each channel across the whole task period. Motion artifacts were visually inspected and detected using the motion detection function ‘hmrMotionArtifactbyChannel’ for the channel-wise signal (tMotion = 0.5 s, tMask = 0.9 s) (Huppert et al., 2009). Channel-wise motion detection and spline correction were performed using a combination of the spline interpolation method and the Savitzky–Golay filter, implemented using the ‘hmrMotionArtifactSpline’ function in the HOMER package (Scholkmann et al., 2010; Yücel et al., 2014). A frame size of 5 and filter order (p) of 0.99 was used for this purpose (Scholkmann et al., 2010; Yücel et al., 2014). Channel data were de-trended to correct for baseline fluctuations and averaged across blocks to increase the signal-to-noise ratio. Relative changes in light intensities were converted into changes in HbO_2_ concentration using the modified Beer–Lambert Law with a path length factor of 6.0 (Scholkmann and Wolf, 2013; Cope et al., 1988). Average haemodynamic responses were estimated around the task onset (60 s after onset) using the ‘hmrDeconvHRF_DriftSS’ function with a short separation of 0 mm (as there was no short separation channels) and the ordinary least squares method (Ye et al., 2009).

### Identification of channel activation

For each group, pre- and post-intervention channel activation was confirmed by comparing the average baseline rest HbO_2_ data sampled over 10 s before task onset (HbO_2Rest_) with average task HbO_2_ data sampled over 110 s starting 10 s after task onset (HbO_2Task_) using the Wilcoxon Signed Ranks test. Channels displaying a statistically significant (*p* < 0.05) increase in HbO_2_ were considered activated.

### Comparisons of activation responses

For each channel and each hemoglobin species, a variable ΔHbO_2_ was computed as follows:


ΔHbO2=HbO2Task−HbO2Rest

For each group, HbO_2_ in each channel was compared pre- and post-intervention using the Wilcoxon Signed Ranks test. Similarly, ΔHbO_2_ in each channel was compared between groups in both pre- and post-intervention suturing sessions using the Mann–Whitney U test.

## Results

### Within-group comparisons

#### Subjective workload and heart rate

There was no significant difference between pre- and post-intervention STAI-6 scores or ΔHR in either group (Table 2).

**Table 2:** Within group comparisons of stress, mental imagery ability and performance.

	Textbook reading			Mental rehearsal		
	Pre-intervention	Post-intervention	*p*-value	Pre-intervention	Post-intervention	*p*-value
STAI-6 score (IQR)	41.7 (37.5)	36.7 (17.5)	0.144^*^	28.3 (14.2)	26.7 (10.0)	0.500^*^
∆HR (IQR)	2.0 (10.8)	0.8 (8.3)	0.192	1.0 (15.8)	−0.5 (13.5)	0.083
Mean LV ± SD	5.28 ± 0.47	4.82 ± 1.50	0.347^†^	5.31 ± 0.97	3.48 ± 1.09	**0.019** ^†^
Mean OPS ± SD	−18.72 ± 120.97	−61.32 ± 99.89	0.221^†^	25.82 ± 51.60	53.10 ± 68.60	0.516^†^
Median TPS (IQR)	6.0 (0.5)	5.8 (0.7)	0.593^*^	6.0 (0.0)	6.0 (0.2)	0.317^*^
MIQ score ± SD	29.00 ± 6.45	29.50 ± 8.50	0.788^†^	36.83 ± 5.78	41.50 ± 4.59	0.097^†^

STAI-6, 6-item state–trait anxiety inventory; ∆HR, change in median heart rate from rest to task (beats per minute); LV, leak volume; OPS, objective performance score; TPS, task progression score; MIQ, mental imagery questionnaire; IQR, interquartile range; SD, standard deviation.

* Wilcoxon signed ranks test.

† Paired samples t-test.

Statistically significant results are in bold.

#### Technical performance

**Figure 3:**
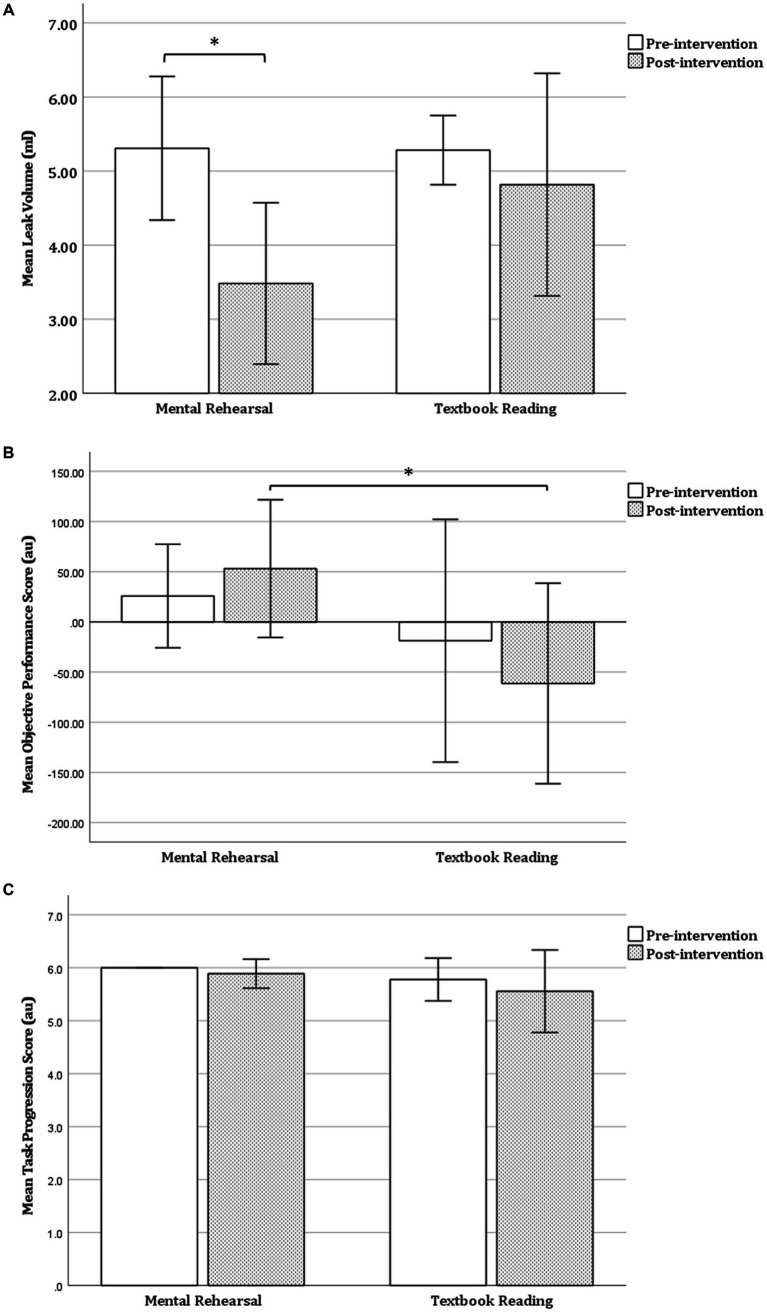
Within-group comparison of task performance. **(A)** Leak volume **(B)** objective performance score and **(C)** task progression score before and after mental rehearsal or textbook reading. Error bars represent 1 standard deviation. Au, arbitrary units; *p < 0.05.

Following MR, there was a significant decrease in LV (mean ± SD: 5.31 ± 0.97 vs. 3.48 ± 1.09, *p* = 0.019), a non-significant increase in OPS (mean ± SD: 25.82 ± 51.60 vs. 53.10 ± 68.60, *p* = 0.516), but no significant change in TPS (*p* = 0.317) (Table 2 and Figure 3). In contrast, TR was not associated with any significant change in OPS (*p* = 0.221), LV (*p* = 0.347), or TPS (*p* = 0.593) (Table 2 and Figure 3).

#### Mental imagery ability

There was a non-significant increase in MIQ score following MR (mean ± SD: 36.83 ± 5.78 vs. 41.50 ± 4.59, *p* = 0.097), but no change in MIQ score following TR (mean ± SD: 29.00 ± 6.45 vs. 29.50 ± 8.50, *p* = 0.788) (Table 2).

#### Prefrontal cortex activation

**Figure 4:**
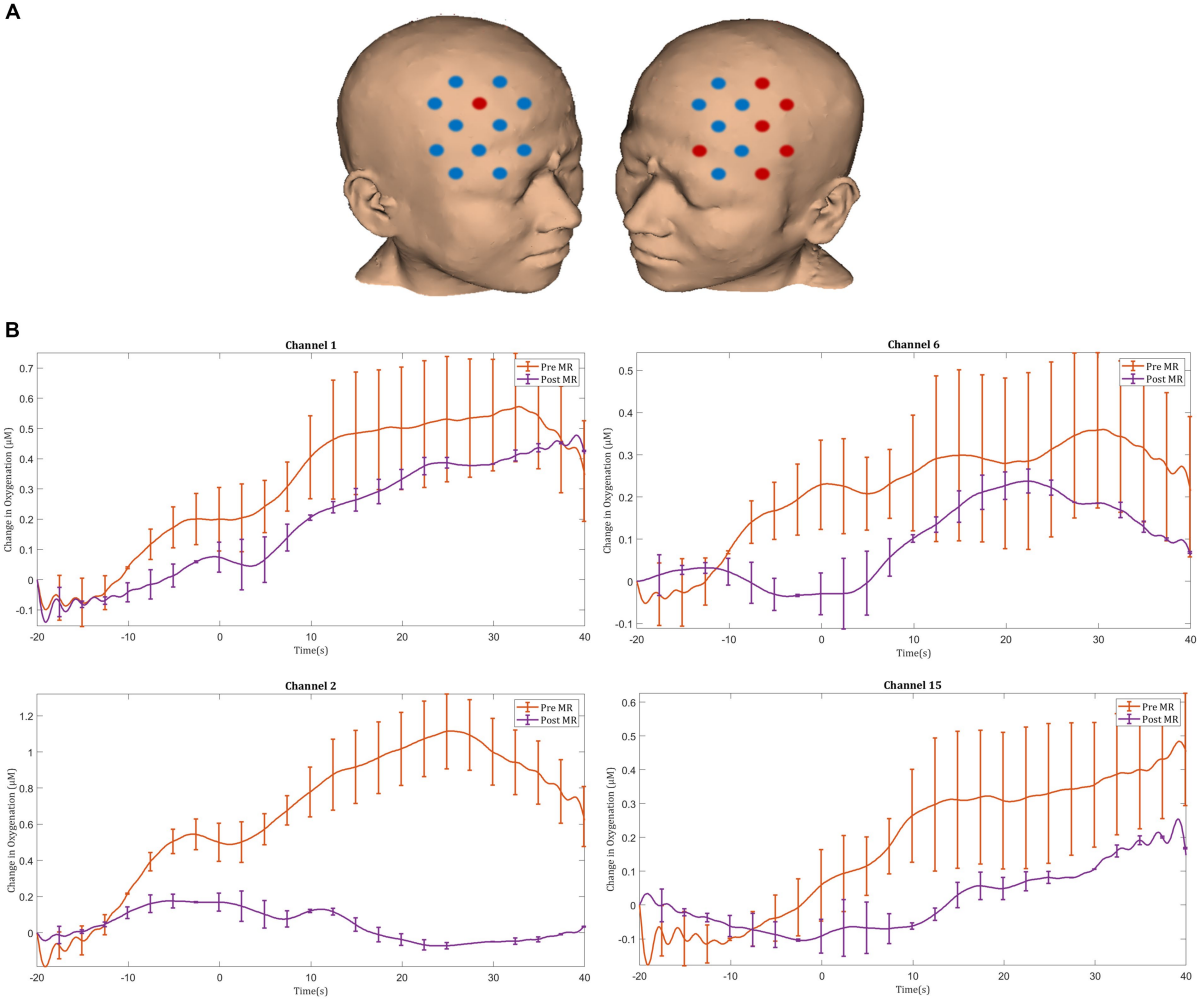
**(A)** Comparison of ΔHbO_2_ between pre- and post-mental rehearsal. Red channels indicate those in which ΔHbO_2_ is greater after mental rehearsal, and blue channels represent those in which ΔHbO_2_ is smaller after mental rehearsal. Channels in which there is a statistically significant difference in ΔHbO_2_ (*p* < 0.05) are circled black. **(B)** Group-averaged time course data from illustrative prefrontal cortical channels demonstrating task-induced change in HbO_2_ concentration pre- and post-mental rehearsal (MR). Task onset occurred at the 0-s time point.

Prior to MR, laparoscopic knot tying was associated with an increase in HbO_2_ concentration in the bilateral PFC, with significant activation responses seen in the right middle frontal gyrus (channel 15). Following MR, significant deactivation responses were observed in the right superior frontal gyrus (channel 22). Following MR there was a trend toward smaller magnitude ΔHbO_2_ responses in the majority of prefrontal channels, however these changes did not reach statistical significance (Figures 4A,B).

**Figure 5:**
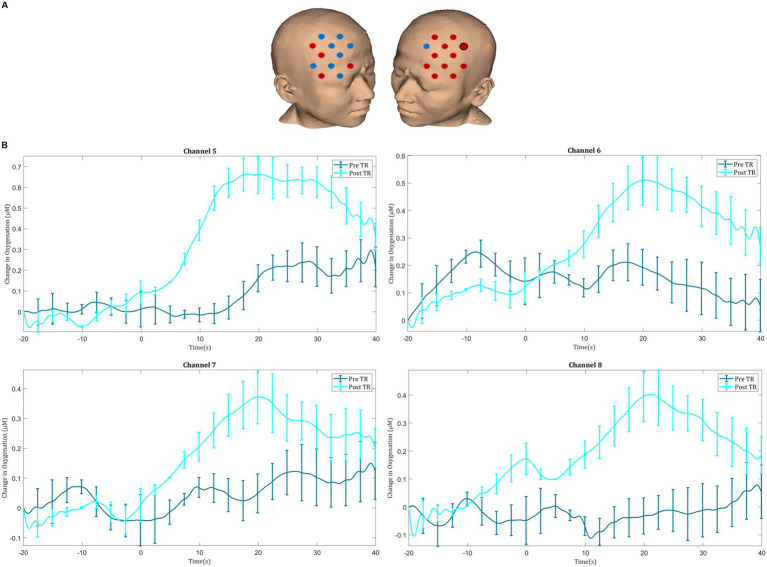
**(A)** Comparison of ΔHbO_2_ between pre- and post-textbook reading. Red channels indicate those in which ΔHbO_2_ is greater after textbook reading, and blue channels represent those in which ΔHbO_2_ is smaller after textbook reading. Channels in which there is a statistically significant difference in ΔHbO_2_ (*p* < 0.05) are circled black. **(B)** Group-averaged time course data from illustrative prefrontal cortical channels demonstrating task-induced change in HbO_2_ concentration pre- and post-textbook reading (TR). Task onset occurred at the 0-s time point.

Before TR, significant activation was seen in the right superior frontal gyrus (channel 20) during laparoscopic suturing. After TR, significant activation was seen in the left middle frontal gyrus (channel 1), left superior frontal gyrus (channel 12), and the right superior frontal gyrus (channel 20). Following TR greater ΔHbO_2_ responses were observed in the left prefrontal cortex, particularly in the left inferior frontal gyrus (channel 9) (Figures 5A,B).

#### Motor cortex activation

**Figure 6:**
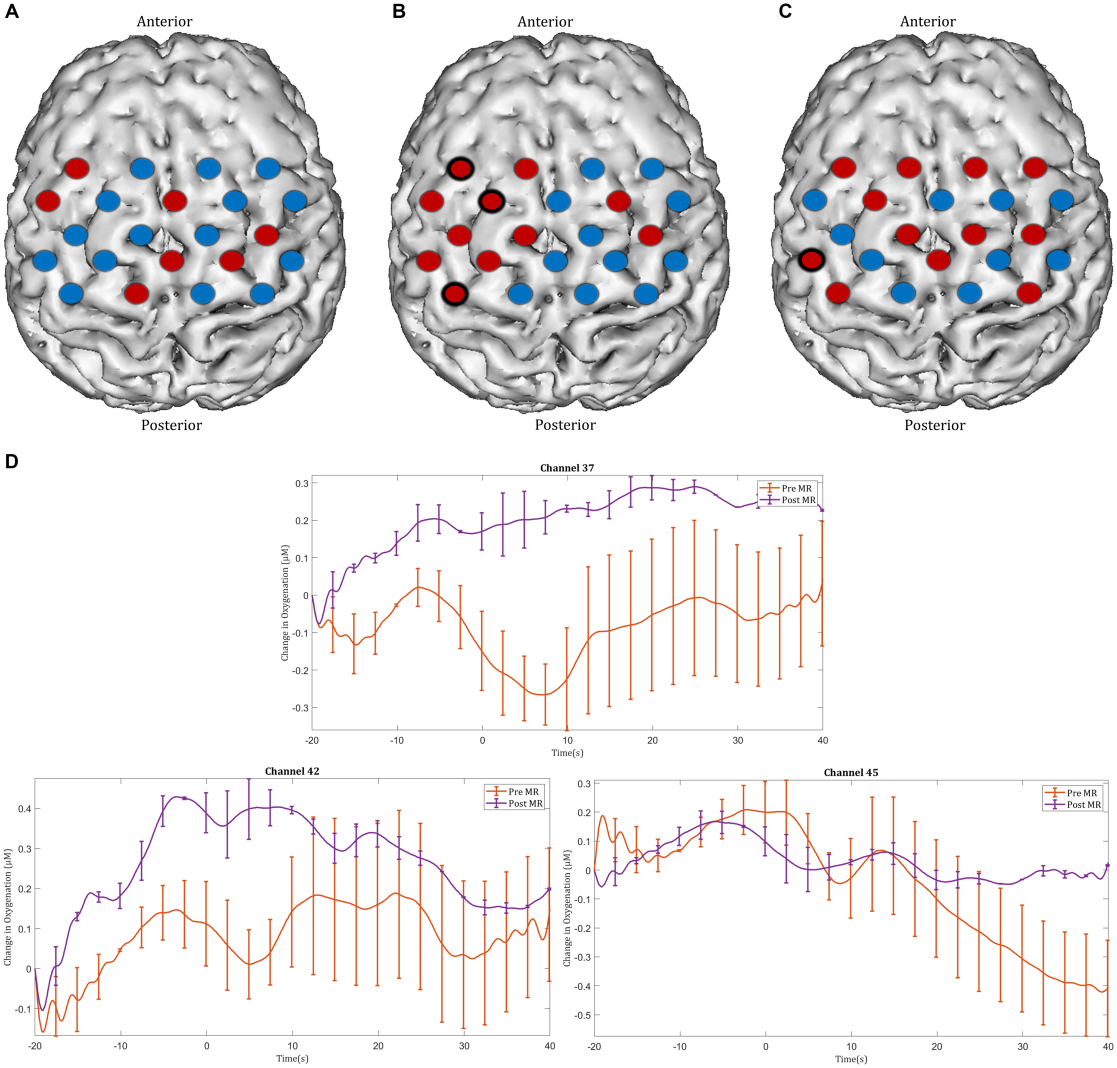
Task-induced changes in HbO_2_ concentration in the motor cortex in **(A)** pre-mental rehearsal and **(B)** post-mental rehearsal. Red channels indicate those in which there was an increase in HbO_2_ from baseline, and blue channels represent those in which there was a decrease in HbO_2_. Channels in which there was a statistically significant change in HbO_2_ concentration (*p* < 0.05) are circled black. **(C)** Comparison of ΔHbO_2_ between pre- and post-mental rehearsal. Red channels indicate those in which ΔHbO_2_ is greater after mental rehearsal, and blue channels represent those in which ΔHbO_2_ is smaller after mental rehearsal. Channels in which there is a statistically significant difference in ΔHbO_2_ (*p* < 0.05) are circled black. **(D)** Group-averaged time course data from illustrative left motor cortical channels demonstrating task-induced change in HbO_2_ concentration pre- and post-mental rehearsal (MR). Task onset occurred at the 0-s time point.

After MR, an increase in HbO_2_ concentration was observed in channels located in the left motor cortex, with significant activation seen in the left middle frontal gyrus (channel 25), the left precentral gyrus (channel 32), and the left postcentral gyrus (channel 41) (Figures 6A,B). Furthermore, greater ΔHbO_2_ activation responses were identified in the left postcentral gyrus (channel 40) following MR (Figures 6C,D).

**Figure 7:**
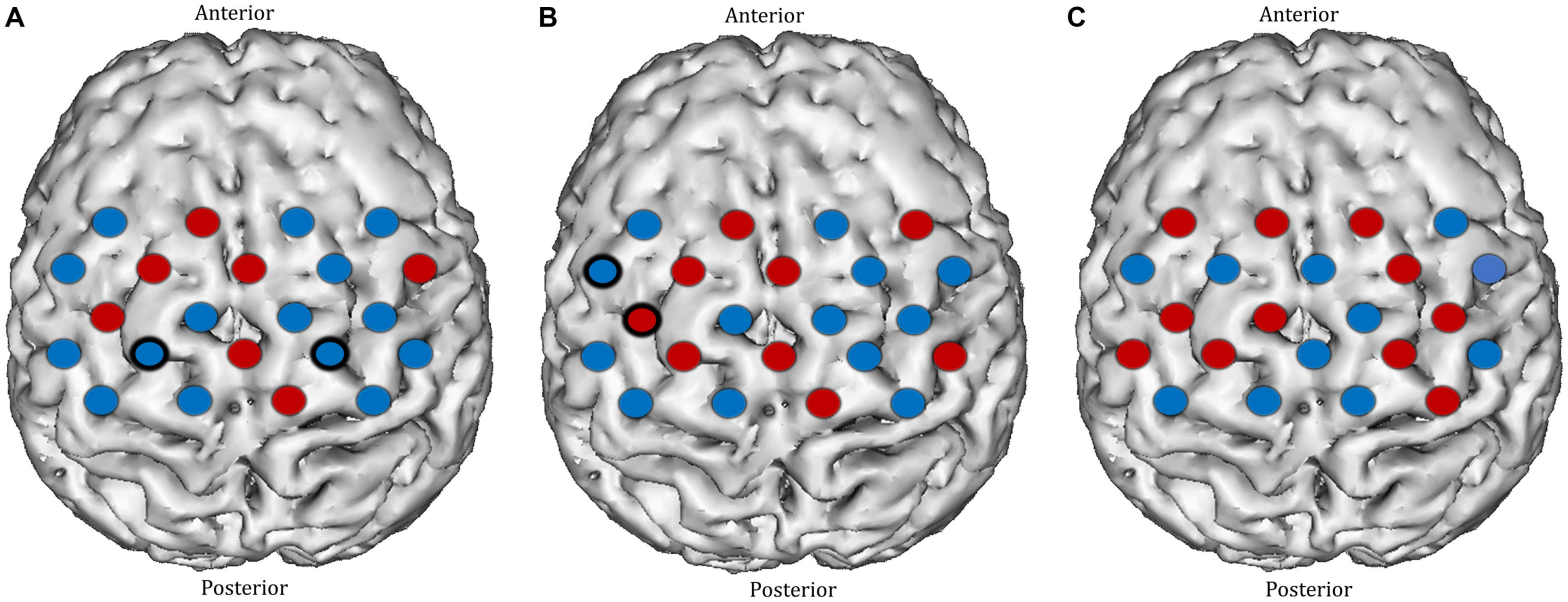
Task-induced changes in HbO_2_ concentration in the motor cortex in **(A)** pre-textbook reading and **(B)** post-textbook reading. Red channels indicate those in which there was an increase in HbO_2_ from baseline, and blue channels represent those in which there was a decrease in HbO_2_. Channels in which there was a statistically significant change in HbO_2_ concentration (*p* < 0.05) are circled black. **(C)** Comparison of ΔHbO_2_ between pre- and post-textbook reading. Red channels indicate those in which ΔHbO_2_ is greater after textbook reading, and blue channels represent those in which ΔHbO_2_ is smaller after textbook reading. Channels in which there is a statistically significant difference in ΔHbO_2_ (*p* < 0.05) are circled black.

Before TR, significant deactivation responses were observed in the left paracentral lobule (channel 35) and the right precentral gyrus (channel 39). After TR, significant activation and deactivation responses were seen in the left precentral gyrus (channels 26 and 33) (Figures 7A,B). Overall, no significant change in ΔHbO_2_ was identified in any motor cortex channels after TR (Figure 7C).

### Between-group comparisons

#### Subjective workload and heart rate

There was no significant between-group difference in STAI-6 score or ΔHR in either the pre-intervention or post-intervention phase (Table 3). Furthermore, there was no difference between groups in overall SURG-TLX score (mean SURG-TLX score ± SD MR vs. TR: 133.17 ± 25.13 vs. 145.00 ± 58.22, *p* = 0.657).

**Table 3:** Between group comparisons of stress, mental imagery ability and performance.

	Pre-intervention			Post-intervention		
	Textbook reading	Mental rehearsal	*p*-value	Textbook reading	Mental rehearsal	*p*-value
STAI-6 score (IQR)	41.67 (37.50)	28.33 (14.17)	0.394^*^	36.67 (17.50)	26.67 (10.00)	0.240^*^
∆HR (IQR)	2.0 (10.8)	1.0 (15.8)	0.707	0.8 (8.3)	−0.5 (13.5)	0.862
Mean LV ± SD	5.28 ± 0.47	5.31 ± 0.97	0.956^†^	4.82 ± 1.50	3.48 ± 1.09	0.109^†^
Mean OPS ± SD	−18.72 ± 120.97	25.82 ± 51.60	0.426^†^	−61.32 ± 99.89	53.10 ± 68.60	**0.043** ^†^
Median TPS (IQR)	6.0 (0.5)	6.0 (0.0)	0.140^*^	5.8 (0.7)	6.0 (0.2)	0.293^*^
MIQ score ± SD	29.00 ± 6.45	36.83 ± 5.78	0.051^†^	29.50 ± 8.50	41.50 ± 4.59	**0.012** ^†^

STAI-6, 6-item state–trait anxiety inventory; ∆HR, change in median heart rate from rest to task (beats per minute); LV, leak volume; OPS, objective performance score; TPS, task progression score; MIQ, mental imagery questionnaire; IQR, interquartile range; SD, standard deviation.

* Mann–Whitney U test.

† Independent samples t-test.

Statistically significant results are in bold.

#### Technical performance

In the pre-intervention session, there were no significant between-group differences in OPS (*p* = 0.426), LV (*p* = 0.956), or TPS (*p* = 0.140) (Table 3 and Figure 3). However, in the post-intervention session, OPS was significantly higher in the MR group compared with the TR group (mean ± SD: 53.10 ± 68.60 vs. −61.32 ± 99.89, *p* = 0.043). No significant between-group differences were observed in the post-intervention session with respect to LV (*p* = 0.109) or TPS (*p* = 0.293) (Table 3 and Figure 3).

#### Mental imagery ability

In the pre-intervention session, there was no significant difference in MIQ scores between the groups (*p* = 0.051). However, in the post-intervention phase, MIQ scores were significantly higher in the MR group compared with the TR group (41.50 ± 4.59 vs. 29.50 ± 8.50, *p* = 0.012) (Table 3).

#### Prefrontal and motor cortex activation

**Figure 8:**
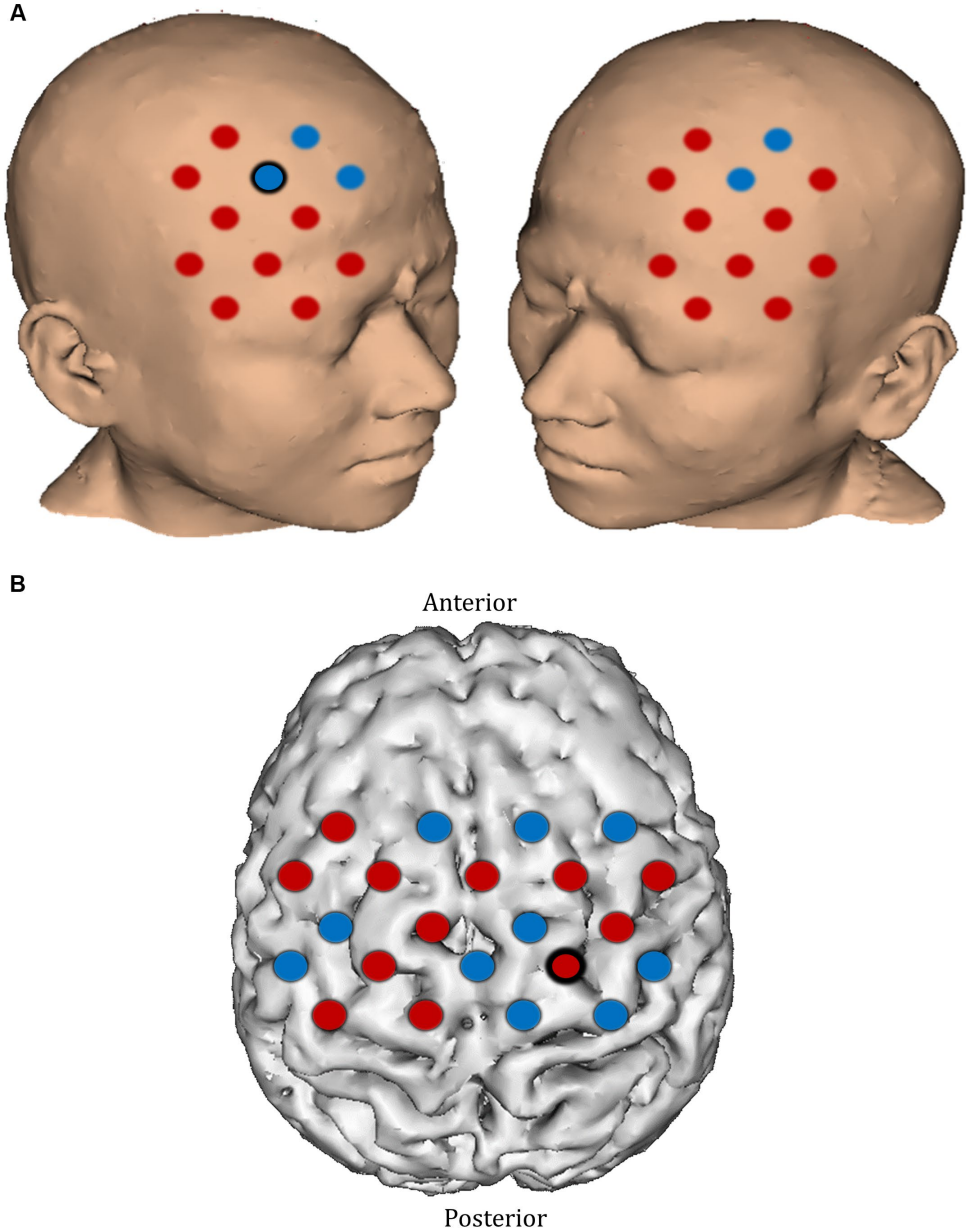
Comparison of ΔHbO_2_ between mental rehearsal and textbook reading in the **(A)** prefrontal and **(B)** motor cortex during the pre-intervention session. Red channels indicate those in which ΔHbO_2_ is greater in the mental rehearsal group compared with the textbook reading group, and blue channels represent those in which ΔHbO_2_ is smaller in the mental rehearsal group compared with the textbook reading group. Channels in which there is a statistically significant difference in ΔHbO_2_ (*p* < 0.05) are circled black.

In the pre-intervention session, apart from a smaller ΔHbO_2_ response in the right superior frontal gyrus (channel 20) and a greater response in the right precentral gyrus (channel 39) in the mental rehearsal group, there were no significant between-group differences in the magnitude of the activation response in any prefrontal or motor cortical channels (Figure 8).

**Figure 9:**
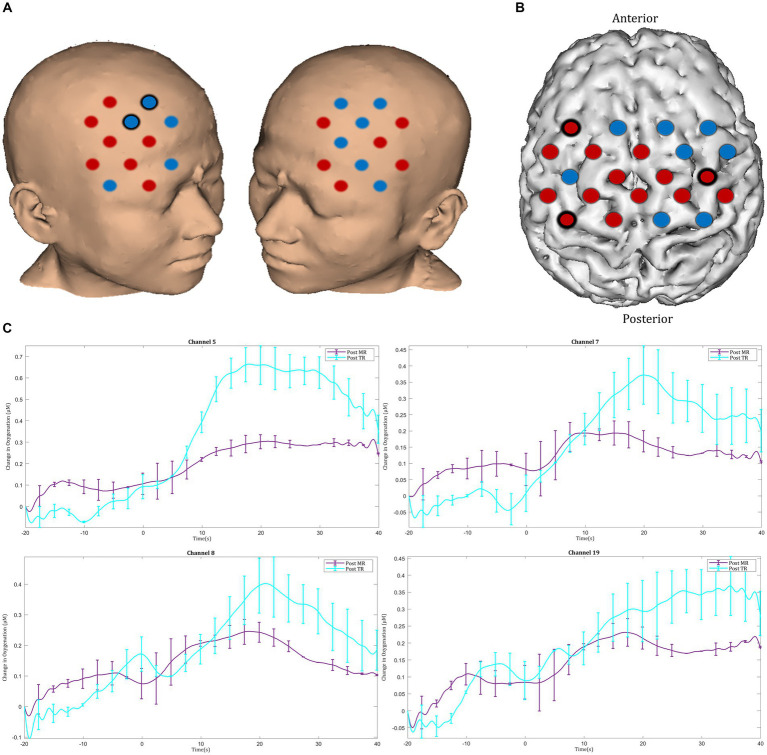

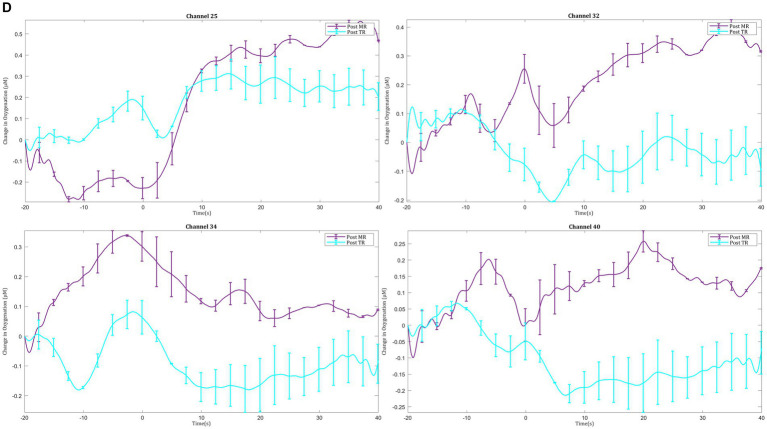
Comparison of ΔHbO_2_ between mental rehearsal and textbook reading in the **(A)** prefrontal and **(B)** motor cortex during the post-intervention session. Red channels indicate those in which ΔHbO_2_ is greater in the mental rehearsal group compared with the textbook reading group, and blue channels represent those in which ΔHbO_2_ is smaller in the mental rehearsal group compared with the textbook reading group. Channels in which there is a statistically significant difference in ΔHbO_2_ (*p* < 0.05) are circled black. Group-averaged time course data from illustrative **(C)** prefrontal and **(D)** left motor cortical channels demonstrating task-induced change in HbO_2_ concentration post-mental rehearsal (MR) and post-textbook reading (TR). Task onset occurred at the 0-s time point.

In the post-intervention session significantly smaller ΔHbO_2_ responses were observed in the MR group in several channels in the right superior frontal gyrus of the prefrontal cortex (channels 20 and 22) compared with the TR group (Figures 9A,C). In the motor cortex, greater ΔHbO_2_ responses were observed in channels located in the left motor cortex in the MR group compared with the TR (e.g., channels 41 and 25) (Figures 9B,D).

## Discussion

This comparative study sought to delineate the effects of mental rehearsal on cortical haemodynamic responses (using fNIRS) and technical ability of surgical trainees performing a laparoscopic suturing task. In line with our hypothesis, mental rehearsal led to attenuated prefrontal responses, greater neural efficiency within the motor cortex, and improvements in technical performance compared with textbook reading.

### Mental rehearsal and surgical performance

In this study, mental rehearsal was found to significantly improve performance on a laparoscopic suturing task. This is congruent with findings from other studies which have also demonstrate enhanced surgical performance following mental rehearsal training (Arora et al., 2011; Cocks et al., 2014; Komesu et al., 2009; Bathalon et al., 2005; Sanders et al., 2008; Immenroth et al., 2007). For example, in study by Sanders et al. (2008) novice surgeons were randomized to receive either further mental rehearsal or engage in textbook reading after which they were asked to open and close a wound on a live anesthetised rabbit (Sanders et al., 2008). The authors found that mental rehearsal was associated with improved technical performance and appeared to facilitate skills transfer from practice to actual surgery better than textbook reading (Sanders et al., 2008). Others have investigated the effects of mental rehearsal on more advanced procedures. For example, Immenroth et al. (2007) randomized surgical trainees into ‘no training,’ ‘practical training,’ or ‘mental rehearsal’ groups (Immenroth et al., 2007). Assessment of technical performance on a simulated laparoscopic cholecystectomy after the assigned intervention showed significant performance gains in the mental rehearsal group, but not in the no training or practical training groups (Immenroth et al., 2007). Similarly, a randomized controlled trial by Arora et al. (2011) demonstrated that surgical novices who received mental rehearsal training in addition to physical practice obtained significantly higher Objective Structured Assessment of Technical Skills (OSATS) scores on a simulated laparoscopic cholecystectomy task compared with those who undertook physical practice alone (Arora et al., 2011). Improvements in technical performance with mental rehearsal have also been observed during cystoscopy (Komesu et al., 2009), crycothyroidotomy (Bathalon et al., 2005) and endovascular surgery (Patel et al., 2012).

Interestingly, certain studies fail to show performance benefit of mental rehearsal during a range of surgical procedures such as laparoscopic suturing and knot-tying (Donnon et al., 2005; Jungmann et al., 2011), pattern cutting (Mulla et al., 2012), carotid endarterectomy (Wetzel et al., 2011), and hysterectomy (Geoffrion et al., 2012). However, these studies have several limitations which may explain the apparent ineffectiveness of MR training interventions. Firstly, none of the studies used any form of ‘manipulation checks’ such as imagery diary exercises or post-intervention interviews that explore participants imagery experience and ensure that subjects are compliant with the mental imagery script (Sevdalis et al., 2013). Indeed, Geoffrion et al. (2012) admit that subjects in the textbook reading arm of the study may have unknowingly mentally rehearsed the procedure as they were reading the relevant textbook chapters (Geoffrion et al., 2012). Secondly, it appears that subjects’ imagery ability was not assessed in any of these studies. This is an important consideration in order to control for differences in imagery ability between control and intervention groups (Sevdalis et al., 2013), and can be achieved by using, for example, the mental imagery questionnaire (MIQ) which can determine individuals’ ability to generate and control images in their mind (Arora et al., 2011). Without manipulation checks or assessment of imagery ability, the internal validity of the results of any mental rehearsal study may be called into question. Finally, there was a prolonged time interval between mental rehearsal and task execution in some of the studies. Evidence suggests that mental rehearsal is most effective when carried out no more than 24 h prior to physical task performance (Sapien and Rogers, 2012). However, in some studies the time lag between mental rehearsal and physical task performance was up to 1 week (Donnon et al., 2005; Mulla et al., 2012), whereas in others the timing was unclear (Jungmann et al., 2011; Wetzel et al., 2011), thereby mitigating any positive effect mental rehearsal may have had on task execution. These potential methodological shortcomings were addressed in the current study. For example, MIQ scores in the mental rehearsal and textbook reading groups were found to be equal at baseline, and the intervention was received immediately prior to physical task execution.

### Mental rehearsal and stress

There was no difference between groups in terms of subjective workload or stress in the current study. This finding is mirrored in other studies which also failed to show that mental rehearsal attenuates stress in surgeons. For example, Wetzel et al. (2011) demonstrated no difference in objective or subjective stress between mental rehearsal and control groups when using a simulated carotid endarterectomy model. Instead, subjects in the mental rehearsal group were found to be using more stress-coping strategies (Wetzel et al., 2011). Similarly, mental rehearsal had no effect on self-reported stress among novice surgeons during a basic surgical skills task (Sanders et al., 2008). The lack of effect of MR on workload and stress observed in the current study would suggest that gains in technical performance observed with MR may be due to its direct impact on surgeons’ learning curves and the underlying neural processes rather than an indirect consequence of reducing cognitive workload.

### Mental rehearsal and prefrontal cortical activity

In the current study, mental rehearsal led to attenuated responses in the prefrontal cortex as well as improved technical performance during laparoscopic suturing, whereas no such changes in activation were observed with textbook reading.

There is wealth of neuroimaging literature highlighting the importance of the prefrontal cortex for novel skill acquisition in surgery (Leff et al., 2007; Leff et al., 2008; Leff et al., 2008; Nemani et al., 2015; Leff et al., 2008; Ohuchida et al., 2009; Shetty et al., 2016; Khoe et al., 2020). For example, Leff et al. (2008) used fNIRS to investigate the neurocognitive mechanisms of task-related expertise in 62 surgeons of varying experience performing five trials of a bimanual open knot-tying task. Technical skill was assessed objectively using time on task, number of movements and instrument pathlength. Whist experienced subjects demonstrated stable technical performance and minimal fluctuation in PFC activity across all five trials, novice surgeons showed significant performance improvement and prefrontal attenuation suggesting that practice-related acquisition of a novel task is associated with a decrease in prefrontal demands (Leff et al., 2008). Similar results have been observed with laparoscopic procedures. Khoe et al. (2020) used fNIRS to examine the variation in PFC activation before and after a laparoscopic training workshop. Novice medical students were randomized into trained and untrained groups. All subjects were shown a basic tutorial video, following which the trained group received additional one-on-one training. Activation responses in the left PFC among the trained group were significantly less than those observed in the untrained group (Khoe et al., 2020). Similarly, Nemani et al. (2018) sought to objectively differentiate surgical skill by assessing patterns of cortical activation during a laparoscopic pattern cutting exercise. Attending surgeons demonstrated decreased PFC activity compared with surgical residents when performing the task on a physical trainer (Nemani et al., 2018).

These studies suggest that in the early phases of motor learning when performance is unrefined and attention-demanding, there is greater recruitment of prefrontal regions (Geoffrion et al., 2012). However, as expertise develops and skills become more automated less demands are placed on executive centers and prefrontal activity diminishes (Leff et al., 2007; Leff et al., 2011). Therefore, the attenuated prefrontal responses that accompanied technical skill improvement in the mental rehearsal group in the current study, may suggest that MR accelerates skill acquisition and expertise development in surgeons at both a motor and cognitive level. Given that mental rehearsal has been shown to activate similar areas of the brain as physical task execution (Moran, 2009; Jeannerod, 2001; Jeannerod, 1995; Kuhtz-Buschbeck et al., 2003; Gerardin et al., 2000; Saiote et al., 2016; Hardwick et al., 2018), it could be hypothesized that MR helps encode a skill on a cognitive level, strengthens central representations of the skill, and facilitates automaticity the same way that physical practice would be expected to do (Sevdalis et al., 2013).

### Mental rehearsal and motor cortical activity

In addition to attenuated prefrontal responses, mental rehearsal led to a spatially more localized increase in activation in the left motor cortex compared with textbook reading—indicative of greater neural efficiency. Given that all subjects in this study were right hand dominant, left lateralisation of motor activity in the mental rehearsal group is likely to genuinely reflect more efficient motor representation during task execution.

There is evidence to suggest that similar neuroplastic changes occur during skills learning. For example, Nemani et al. (2018) used fNIRS to capture cortical haemodynamic responses over the PFC, SMA and M1 to classify surgical expertise during a laparoscopic pattern cutting task. Novice surgeons were shown to have significantly greater activation in the PFC and less activation in the left medial M1 and SMA compared with expert surgeons (Nemani et al., 2018). These findings have been confirmed by longitudinal studies which have delineated changes in motor cortical activity as expertise develops over time (Floyer-Lea and Matthews, 2005; Karni et al., 1995; Ma et al., 2010). Floyer-Lea and Matthews (2005) sought to identify changes in cortical activation that occur with short term (fast) learning during which performance improves rapidly, and long term (slow) learning during which performance gains are incremental. Using fMRI, the authors showed that during short-term learning of an isometric force task, activity in the PFC and primary motor cortex decreased, whereas with long term learning (3 weeks), increased activity was observed in the primary motor cortex (Floyer-Lea and Matthews, 2005). In line with these findings, Karni et al. (1995) trained subjects in a motor sequence task for 3 weeks and found a progressive increase in activation of the primary motor cortex. These findings suggest that over time, there is an enlargement of the motor cortical representation a learned skill, which may underlie long-term skill retention and enable finer motor control (Ungerleider et al., 2002; Dayan and Cohen, 2011).

Neural efficiency refers to patterns of more spatial localized or less intense brain activity when performing a motor skill (Neubauer and Fink, 2009). Many studies have shown that brain activation in expert athletes is more spatially localized compared with non-experts. For example, Chang et al. (2011) compared the activation maps of elite archers and non-archers during mental rehearsal using fMRI. In non-archers, a wide area of activation was observed and included premotor, SMA, inferior frontal region, basal ganglia and cerebellum. In contrast, activation was localized to only the SMA in expert archers (Chang et al., 2011). Similarly, Milton et al. (2007) used fMRI to compare brain activation during the pre-shot routine of novice and expert golfers and found a more focused and efficient organization of task-related neural networks among expert golfers. In contrast, novices exhibited a broader area of activation implying a difficulty in filtering out task-irrelevant information (Milton et al., 2007).

In sum, motor skill learning is associated with a redistribution of cortical activity from anterior to posterior regions (Neubauer and Fink, 2009; Kelly and Garavan, 2005). Specifically, as expertise develops prefrontal cortical activity attenuates, interpreted as less consumption of attentional resources as a skill is internalized and becomes automated (Leff et al., 2011). Concurrently, an increase in activity in motor regions is observed suggesting greater recruitment of additional motor units into a local network that represents that acquired skill (Ungerleider et al., 2002). Furthermore, this increase in activity in motor regions is more spatially localized to task-relevant cortical regions suggesting a greater degree of neural efficiency as learning progresses. In the current study, mental rehearsal led to similar neuroplastic changes in prefrontal (decreased activity) and motor regions (increased and spatially more localized activity), which suggests that mental rehearsal facilitates skill acquisition by accelerating the neuroplastic changes that accompany skills learning.

### Limitations

There are several limitations to the current study which need to be acknowledged. Firstly, the number of participants was relatively low which increases the risk of type 1 and/or type 2 errors. However, a sample size estimation was not feasible as there have been no previous studies comparing mental rehearsal and textbook reading in a surgical setting in order to perform a pre-hoc power calculation. Indeed, studies investigating the effect of mental rehearsal on cortical activation in other domains have not incorporated sample size calculations and the cohort size in our study is comparable to these studies (Nyberg et al., 2006; Moriya and Sakatani, 2017). Furthermore, the current work is a hypothesis-generating study, data from which could be used for a sample size calculation for a larger confirmatory study.

Secondly, there may be a selection bias in the recruitment of study participants. Although all general surgical residents within a postgraduate training region were invited to participate, it is possible that only residents who felt confident in their laparoscopic knot-tying ability agreed to enroll. The subspecialty interest of participants was not recorded, and there may have been a disproportionate number of residents with a specialist interest in surgical disciplines in which laparoscopic skills are a fundamental part of training (e.g., upper gastrointestinal surgery). Therefore, the neuroergonomic and performance benefits of mental rehearsal may not be representative of the wider surgical community. Furthermore, generalisability of the study findings to other surgical procedures cannot be assumed. Empirical data is required to ascertain whether mental rehearsal can have similar effects on performance and brain behavior during other types of surgical skill.

Thirdly, whilst mental imagery ability was found to be comparable between the two groups at baseline, manipulation checks to determine whether subjects are adhering to the MR script were not carried out. Future studies can address this shortcoming by conducting post-intervention interviews or collecting qualitative data from imagery diaries.

Fourthly, short separation channel data was not collected which would have provided an indication as to whether the measured cortical activations were contaminated by superficial haemodynamic signals. However, analysis of heart rate data demonstrated that there was no significant change in the systemic physiological response within or between groups which would suggest that the observed cortical haemodynamic signals reflect genuine underlying activation responses.

Finally, we did not investigate whether the effects of mental rehearsal on cortical activation are retained in the long-term. This would be the focus of future work to provide supportive evidence for incorporating mental skills training in the surgical curriculum. Nonetheless, the short-term neuroergonomic benefits of mental rehearsal which this study investigates are equally important to appreciate since, in the real-world setting, surgeons usually mentally visualize the critical steps of the procedure, often with the aid of pre-operative imaging, just prior to starting an operation.

## Conclusion

Mental rehearsal is associated with neuroplastic changes that accompany skills learning and expertise development, as well improved technical performance during a laparoscopic surgical task. Specifically, it is associated with attenuated prefrontal activation and greater neural efficiency in motor regions, suggesting reduced attentional demands, greater task automaticity and encodement of motor skill at a cognitive level. The current study findings suggest that mental rehearsal may be used as an adjunct to traditional training strategies to enhance skill acquisition among trainee surgeons.

## Data Availability

The raw data supporting the conclusions of this article will be made available by the authors, without undue reservation.
