# Supplementary material for: Investigating the impact of mental rehearsal on prefrontal and motor cortical haemodynamic responses in surgeons using optical neuroimaging
Source: Front Hum Neurosci. 2024 Oct 21;18:1386005. doi: 10.3389/fnhum.2024.1386005 (PMC11532121; doi:10.3389/fnhum.2024.1386005)
Supplement: Supplementary file 1 [file Data_Sheet_1.docx]

**Supplementary Text S1. Mental Rehearsal Script**

I move my right needle-holder towards the suture needle which I can see lying near the Penrose drain. I pick up the needle two thirds of the way along its length, holding it in a ‘smile shape’ and approach the drain aiming to pierce the entry and exit dots on either side of the drain. Using my left needle-holder, I hold the drain to create tension. As I puncture the drain with the needle, I supinate my wrist to minimise tissue damage and ensure the path of the needle is smooth. I can feel the resistance of the drain. When I see the tip of the needle poking out of the other side of the drain, I release the needle from my right needle-holder and pick up the tip with the same hand in the pronated position. I supinate my right hand so that when I pull the needle out it is held in a ‘frown shape’. I place my left needle-holder on the drain to help pull the suture through. I now have a short end and a long end of the suture. To perform the double throw, I use the right needle-holder to wrap the suture around the left needle-holder twice and use the left needle-holder to grab the short end of the suture. I move my hands in opposite directions to tighten the knot. To perform the first single throw, I swap the needle to my left hand and wrap the suture around the right needle-holder once. I pick up the short end of the suture with my right needle holder, again moving my hands in opposite directions to tighten the suture. For the final single throw, I swap the needle back to my right needle-holder and repeat the movement. I then hold both suture ends up and under tension with my left needle-holder and replace my right needle-holder with a pair of laparoscopic scissors which I use to cut the two threads of the suture short.

**Supplementary Figure S1. Mental Imagery Questionnaire**

| **1. How ready or ‘energised’ do you feel to perform a laparoscopic knot-tying task?** | | | | | | | | |
| --- | --- | --- | --- | --- | --- | --- | --- | --- |
| Not at all | 1 | 2 | 3 | 4 | 5 | 6 | 7 | Very |
| **2. How confident do you feel about performing a laparoscopic knot-tying task?** | | | | | | | | |
| Not at all | 1 | 2 | 3 | 4 | 5 | 6 | 7 | Very |
| **3. How well do you think you can perform a laparoscopic knot-tying task compared to others at your stage of training?** | | | | | | | | |
| Not at all | 1 | 2 | 3 | 4 | 5 | 6 | 7 | Very |
| **4. How helpful is the activity you have just been doing in preparing you to perform a laparoscopic knot-tying task?** | | | | | | | | |
| Not at all | 1 | 2 | 3 | 4 | 5 | 6 | 7 | Very |
| **5. How easily can you ‘see’ yourself performing a laparoscopic knot-tying task?** | | | | | | | | |
| Not at all | 1 | 2 | 3 | 4 | 5 | 6 | 7 | Very |
| **6. How vivid or clear are the images of the laparoscopic knot-tying task in your mind?** | | | | | | | | |
| Not at all | 1 | 2 | 3 | 4 | 5 | 6 | 7 | Very |
| **7. How easily can you ‘feel’ yourself performing the laparoscopic knot-tying task?** | | | | | | | | |
| Not at all | 1 | 2 | 3 | 4 | 5 | 6 | 7 | Very |
| **8. How easily would you be able to talk someone through the steps of the laparoscopic knot-tying task?** | | | | | | | | |
| Not at all | 1 | 2 | 3 | 4 | 5 | 6 | 7 | Very |
